# Sucrose-Responsive Intercommunicated Janus Nanoparticles Network

**DOI:** 10.3390/nano11102492

**Published:** 2021-09-24

**Authors:** Sandra Jimenez-Falcao, Daniel Torres, Paloma Martínez-Ruiz, Diana Vilela, Ramón Martínez-Máñez, Reynaldo Villalonga

**Affiliations:** 1 Nanosensor & Nanomachines Group, Department of Analytical Chemistry, Faculty of Chemistry, Complutense University of Madrid, 28040 Madrid, Spain; sandraji@ucm.es (S.J.-F.); danitorres.1995@gmail.com (D.T.); palmarti@quim.ucm.es (P.M.-R.); divilela@ucm.es (D.V.); 2 Instituto Interuniversitario de Investigación de Reconocimiento Molecular y Desarrollo Tecnológico (IDM), Universitat Politècnica de València, Universitat de València, Camino de Vera s/n, 46022 Valencia, Spain; Ramon.rmaez@qim.upv.es; 3 Research Mix Unit of Disease Mechanisms and Nanomedicine UPV-CIPF, Universitat Politècnica de València, Centro de Investigación Príncipe Felipe, Eduardo Primo Yúfera, 3, 46012 Valencia, Spain; 4 Networking Biomedical Research Centre in Bioengineering, Biomaterials and Nanomedicine, (CIBER-BBN), Av. Monforte de Lemos 3-5, Pabellón 11, Planta 0, 28029 Madrid, Spain

**Keywords:** network, Janus particles, enzymatic control, nanodevices, intercommunication

## Abstract

Inspired by biological systems, the development of artificial nanoscale materials that communicate over a short distance is still at its early stages. This work shows a new example of a cooperating system with intercommunicated devices at the nanoscale. The system is based on the new sucrose-responsive Janus gold-mesoporous silica (Janus Au-MS) nanoparticles network with two enzyme-powered nanodevices. These nanodevices involve two enzymatic processes based on invertase and glucose oxidase, which are anchored on the Au surfaces of different Janus Au-MS nanoparticles, and N-acetyl-L-cysteine and [Ru(bpy)_3_]^2+^ loaded as chemical messengers, respectively. Sucrose acts as the INPUT, triggering the sequential delivery of two different cargoes through the enzymatic control. Nanoscale communication using abiotic nanodevices is a developing potential research field and may prompt several applications in different disciplines, such as nanomedicine.

## 1. Introduction

Communication is the act of transmitting information. Every communication involves at least one sender, a message, and a recipient. Although it may seem simple, communication is actually a very complex process. Among communication types, molecular communication, inspired by biological systems, uses molecules as an information medium. Molecular communication allows biologically and artificially created micro- or nanoscale entities to communicate over short distances [1].

Nowadays, nanotechnology provides a new set of tools, allowing the engineering community to design nanomachines that open numerous application opportunities. Synthetic nanomachines are basic functional nanodevices able to perform very simple tasks. Despite the promising development of biomimetic artificial nanomachines capable of carrying out different functions similar to nature nanoscale machines found in living cells, an individual nanodevice is highly restricted by the limited energy, processing, and communication range [2,3,4]. Therefore, the nanonetwork, the network at the nanoscale, extends the capabilities of a single nanodevice through the cooperation and information sharing. As such, the nanonetwork has become a powerful technological tool, enabling the detection, acquisition, and monitoring of physical magnitudes in application scenarios which were previously unconceivable [5]. Communication between nanoscale devices is an area of considerable importance, as it is essential that future devices are able to form nanonetworks and realize their full potential [6]. Despite the great interest that this topic has gained, communication between abiotic nanodevices has scarcely been studied [7]. Tomasi et al. reported the first experimental and theoretical study on the propagation of the Belousov–Zhabotinsky (BZ) oscillating reaction as a source of chemical information encapsulated in liposomes, and observed that chemical information is transmitted either by the direct transfer of redox active species and/or interfacial electron transfer [8]. Gimenez et al. designed the first advanced cooperative system containing a hierarchically organized community of different nanoparticles that can communicate through chemical messengers. To this end, the researchers prepared a system of three different capped mesoporous silica nanoparticles (C-MSNPs) loaded with three different connected messengers, respectively. The system was activated by an enzyme capable of hydrolyzing the oligosaccharide layer of one of the three C-MSNPs, thus triggering the cascade reaction through the delivery of the first messenger [9]. Recently, we reported different models of communicating systems based on enzyme-controlled Janus gold-mesoporous silica (Janus Au-MS) nanoparticles. These nanosystems contain a mesoporous face, which is loaded with a cargo (messenger) and capped with molecular gates that can be opened in the presence of a specific stimulus. The nanosystems also contain a gold face that is functionalized with enzymes. The first work mimicked an interactive model of communication, where two nanosystems cooperated with each other [10]. In this way, a sender nanoparticle received a stimulus/INPUT and encoded a message for a receiver nanoparticle that interpreted the message and returned feedback to the first nanoparticle. Inspired by this interactive model of communication at the micro/nanoscale, our research group has reported several works [11,12]. De Luis et al. reported a circular model of a multicomponent communication network of nanoparticles based on the exchange of chemical messages [12]. Based on the cyclic regulation of the metabolic processes observed in biological reaction networks, we developed a communication system which consisted of three enzyme-controlled Janus Au-MS nanoparticles which displayed a double receiver-sender behavior [13,14]. Nanodevice 1 was loaded with a fluorescent dye [Ru(bpy)_3_]Cl_2_ which was released at the end of the cycle, indicating the success of the communication system. Briefly, lactose, as enzymatic substrate of the enzyme-nanodevice 1, acted as the external INPUT, triggering the cyclic communication through the production of galactose. Subsequently, galactose was sensed by enzyme-nanodevice 2, which produced H_2_O_2_, opening the capped-mesoporous silica through complex formation and inducing the release of a benzoate derivate in the same device. Enzyme-nanodevice 3 identified the benzoate derivate and transformed it in benzoic acid, inducing a pH drop and, consequently, opening the pH responsive supramolecular nanovalve and releasing reductive species previously loaded in mesoporous face from nanodevice 3. Finally, the reductive species opened the redox nanovalve from nanodevice 1, releasing [Ru(bpy)_3_]^2+^ providing a fluorescent signal.

Regarding the communication of abiotic nanodevices with living systems, we reported a stimuli-responsive interactive paradigm of communication between yeasts and enzyme-controlled Janus Au–MS nanoparticles [11]. In this work, sucrose was used as the INPUT signal, triggering the molecular communication cascade through its detection and transformation into fructose and glucose by the living yeast *Saccharomyces cerevisiae*. The produced glucose, chemical messenger 1, was sensed by glucose oxidase (GOD) on the gold surface of the abiotic nanodevices. This led to the production of gluconic acid, which locally dropped the pH level, thus inducing the opening of the molecular gates of the mesoporous face from the same nanodevice. The opening of the molecular gates on the nanodevices provoked the release of the loaded antibiotic (chemical messenger 2), which was sensed by the microorganism, activating the expression of green fluorescent protein and, thus, generating fluorescent signal. 

**Scheme 1:**
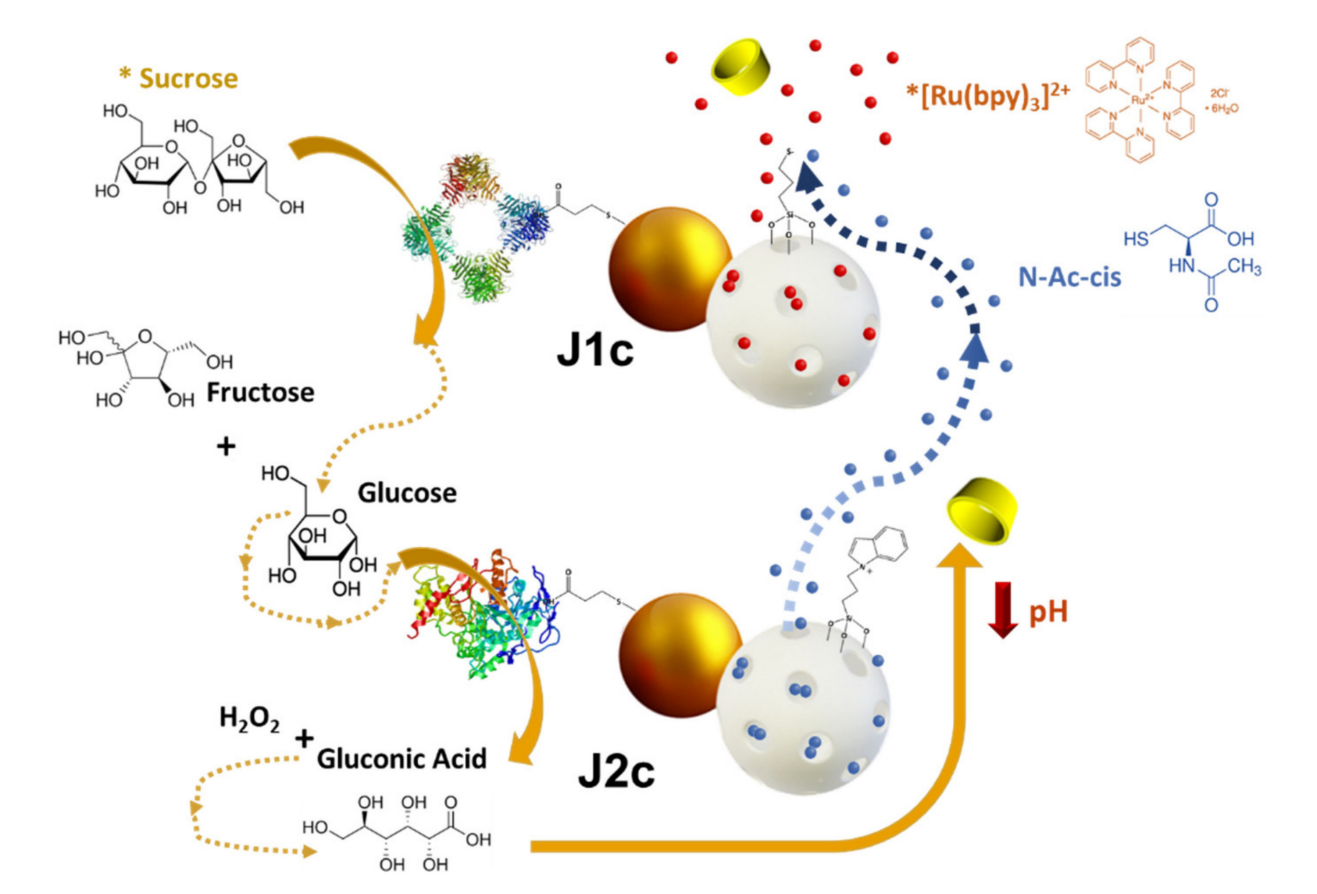
Schematic representation of the performance of the intercommunicated Janus nanoparticle network (J1c and J2c) in presence of sucrose.

Considering this last work, in this study, we designed and developed a sucrose-responsive intercommunicated nanonetwork based on the exchange of chemicals using exclusively abiotic nanodevices. To this end, the nanonetwork systems consisted of two enzyme-controlled Janus gold-gated mesoporous silica nanoparticles which had double roles (receiver-sender). As described in Scheme 1, the mesoporous face from enzyme-nanodevice 1 (J1c) was loaded with [Ru(bpy)_3_]^2+^ and capped with cyclodextrin through the disulfide-bond, which acted as a gatekeeper, whereas the enzyme invertase was anchored to the gold face. In nanoparticle 2 (J2c), the enzyme GOD was immobilized on the Au face, while the mesoporous silica was loaded with N-acetyl-L-cysteine and capped with a responsive supramolecular nanovalve consisting of an inclusion complex between a benzimidazole moiety and β-cyclodextrin. Sucrose was used as the INPUT signal, triggering the molecular communication cascade through its detection and transformation into fructose and glucose by J1c. The produced glucose was sensed by GOD at J2c, producing gluconic acid which locally dropped the pH level and induced the opening of the molecular gates of the mesoporous face from the same nanodevice. The opening of the molecular gates on J2c provoked the release of the loaded N-acetyl-L-cysteine, which carried out the cleavage of the disulfide bond on the mesoporous face of J1c, resulting in the pore opening and [Ru(bpy)_3_]^2+^ delivery. Therefore, the sucrose-responsive-intercommunicated nanonetwork can be monitored by UV-vis.

## 2. Materials and Methods

### 2.1. Materials

Saccharose, cetyltrimethylammonium bromide (CTAB), sodium hydroxide (NaOH), tetraethoxysilane (TEOS), tetrachloroauric(III) acid trihydrate (HAuCl_4_·3H_2_O), trisodium citrate, (3-mercaptopropyl)trimethoxysilane, (3-iodopropyl)trimethoxysilane, tris(2,2′-bipyridyl)dichlororuthenium(II), 3-mercaptopropionic acid, sulfonate-β-cyclodextrin, β-cyclodextrin, benzimidazole, triethylamine, N-acetyl-L-cysteine, N-(3-dimethylaminopropyl)-N′-ethylcarbodiimide hydrochloride (EDC), N-hydroxysuccinimide (NHS), glucose oxidase (GOD), paraffin wax, ethanol, methanol, chloroform, acetonitrile anhydrous (AcN), toluene, glacial acetic acid (AcH), anhydrous DMF, 2,2′-azino-bis(3-ethylbenzothiazoline-6-sulfonic acid (ABTS), horseradish peroxidase (HRP) and phosphate buffer (PB) (0.1 M, pH 7.4) were used in this study.

### 2.2. Equipment

Powder X-ray diffraction (PXRD), transmission electron microscopy (TEM), N_2_ adsorption desorption isotherms, UV-visible and fluorescence spectrophotometry, dynamic light scattering (DLS), and thermogravimetric analysis (TGA) were employed for materials characterization. PXRD was performed with an X’Pert MRD diffractometer (Malvern, UK) using radiation at low and high angles. TEM measurements were performed with a JEOL JEM-2100 microscope. DLS studies were performed using a ZetaSizer Nano ZS (Malvern, UK). N_2_ adsorption-desorption isotherms were recorded on a Micromeritics TriStar II Plus automated analyzer. FT-IR spectra were obtained from KBr discs using a Nicolet Nexus 670/870 spectrometer. TGA was carried out using a thermogravimeter SDT-Q600 (TA Instruments, EE.UU). Spectrophotometric measurements were performed using an Ultrospec 8000 UV/VIS spectrophotometer (General Electric, EEUU).

### 2.3. Preparation of Janus Au-MS Nanoparticles (J)

Preparation of the Mesoporous Silica Nanoparticles (MSNPs) [15]: First, 1 g of CTAB was dissolved in 480 mL of water at 60 °C under vigorous magnetic stirring. Then, the NaOH solution (8% *v*/*v*) was added, and the temperature was increased. When the mixture reached 80 °C, 5 mL of TEOS was added dropwise to the solution, and the temperature was kept constant for 2 h. Afterward, the mixture was allowed to cool down. The produced white solid was filtered, washed (water and methanol), and dried on the stove at approximately 70 °C. Finally, to remove the organic template, the solid (as-synthesized MCM-41) was calcinated at 550 °C for 5 h.

Preparation of the Gold Nanoparticles (AuNPs) [16,17]: First, 100 mL of a 0.3 mM HAuCl_4_·3H_2_O solution was heated to boiling. Then, 5 mL of a 39 mM trisodium citrate solution was added, and the mixture was allowed to react for 10 min. The brilliant red solution obtained was cooled to room temperature. This process was repeated four times to obtain 400 mL of the AuNPs solution.

Preparation of the Janus Mesoporous Silica (Janus Au-MSNPs) Nanoparticles: The preparation of Janus Au-MS Nanoparticles followed the previous protocol described by our group [18]. Briefly, 200 mg of MSNPs was dispersed in 10 mL of 6.7% (*v*/*v*) ethanol aqueous solution. Then, 208 μL of 0.05M CTAB was added, and the mixture was heated. When the water bath reached 75 °C, 1 g of paraffin wax was added and consecutively melted. Afterward, the mixture was vigorously stirred at 25,000 rpm for 10 min using an Ultra-Turrax T-10 homogenizer (IKA, Germany). The resulting emulsion was further stirred through magnetic mixing for 1 h at 75 °C. Then, the emulsion was cooled to room temperature. The Pickering emulsion was mixed with 10 mL of methanol and treated with 200 μL of (3-mercaptopropyl)trimethoxysilane for 3 h under magnetic stirring. Next, the silanized product was filtered, washed three times with methanol, dispersed in 75 mL of methanol, and incubated with 400 mL of the previously prepared AuNP solutions overnight under magnetic stirring. The resulting mixture was filtered and exhaustively washed with methanol. The solid obtained was suspended in methanol and washed with chloroform/methanol (1:1, 2:1, 3:1) and chloroform, successively, to solubilize the paraffin. Finally, the Janus Au-MS Nanoparticles were dried at 70 °C and kept in desiccators.

### 2.4. Preparation of Janus Au-MS Nanoparticles 1 (J1b)

To synthesize J1b, 100 mg of Janus Au-MS Nanoparticles was first suspended in 2 mL of AcN and treated with 100 μL of 3-mercaptopropionic acid for 1h under magnetic stirring to protect the gold face. After washing with AcN, the solid was suspended in 2 mL of AcN. Then, 100 μL of 3-mercaptopropiltrimethoxisilane was added. The mixture was stirred for 12 h at room temperature. Then, the solid was centrifuged and washed several times in AcN. The solid was suspended in 5 mL of the same solvent. Next, to load the dye into the MS face, 60 mg of tris(2,2′-bipyridyl)dichlororuthenium(II) hexahydrate was added to the previous particle suspension in a round-bottom flask connected to a Dean–Stark trap under an Ar atmosphere. First, the mixture was heated at 120 °C to collect the adsorbed water in the trap through the distillation of the solvent. Then, the mixture was stirred for 24 h at room temperature to load the dye into the MS-face pores. The resulting solid was centrifuged and kept in desiccators. The prepared particles were suspended in 2 mL toluene and treated with 3 mL of potassium tert-butoxide solution in the same solvent (33.3 g/L) under magnetic stirring for 15 min. After this step, the solid was washed in toluene and suspended in 2 mL of DMF. The suspension was treated with 50 mg of the sulfonate-β-cyclodextrin at room temperature for 12 h. The prepared product was filtered under vacuum and washed with anhydrous DMF, DMF/AcN (1:1) and AcN/glacial AcH (1% *v*/*v*), successively. After drying, J1b was washed with 0.1 M PB, pH 7.4. Finally, the resulting particles were centrifugated and dried at 70 °C and kept in desiccators.

Tris(2,2′-bipyridyl)ruthenium(II) content was quantified by incubating 5 mg of solid J1 in 2 mL of 1 M NaOH for 1 h. The resulting solution was centrifuged, and the absorbance at 454 nm was measured. In parallel, solutions of different concentrations of tris(2,2′-bipyridyl)ruthenium(II) in 1 M NaOH were treated under the same conditions to further construct a calibration plot for the quantitative determination of the dye. All determinations were performed in triplicate.

### 2.5. Preparation of Janus Au-MS Nanoparticles 2 (J2b)

To synthesize J2b, 100 mg of Janus Au-MS Nanoparticles were first treated to protect the gold face as described previously for J1b. After washing with AcN, the solid was suspended in 5 mL of AcN. Then, 200 μL of (3-iodopropyl)trimethoxysilane was added. The mixture was stirred for 5.5 h at room temperature. Then, the solid was centrifuged and washed several times in toluene. The solid was resuspended in a saturated solution of benzimidazole/triethylamine (1:3) using warm toluene as a solvent. The suspension was refluxed and stirred for 72 h. The resulting solid was filtered off, washed with 40 mL of AcN, and dried at 70 °C overnight. Then, the previous obtained product was resuspended in 4 mL a saturated solution of N-acetyl-L-cysteine (1.23 M), using phosphate buffer (PB, 0.1 M, pH 7.4) as a solvent, and stirred for 12 h. Afterward, 5 mL of a saturated solution of β-cyclodextrin in PB was added, and the mixture was stirred for 12 h at room temperature. The resulting solid, J2b, was centrifuged and washed in PB. 

### 2.6. Preparation of J1c and J2c 

Preparation of J1c: First, 10 mg of J1b was treated with a solution of 7.5 mg of NHS and EDC, respectively, in PB for 1 h under magnetic stirring. Then, the nanoparticles were washed and incubated with 2 mL of invertase solution in PB (3 mg/mL) for 12 h at 4 °C. The J1c was washed and stored in PB at 4 °C.

Preparation of J2c: First, 10 mg of J2b was treated with a solution of 7.5 mg of NHS and EDC, respectively, in PB for 1 h under magnetic stirring. Then, the nanoparticles were washed and incubated with 2 mL of GOD solution in PB (3 mg/mL) for 12 h at 4 °C. The J2c was washed and stored in PB at 4 °C.

The immobilization of the enzymes was confirmed by the quantification of the amount of protein using the Bradford method [19] and running an enzyme activity assay for the solid J1c and J2c, respectively. On the one hand, the method used to test GOD activity is based on the oxidation of glucose by GOD, which produces gluconic acid and hydrogen peroxide. Then, hydrogen peroxide reacts with ABTS (2,2′-azino-bis(3-ethylbenzothiazoline-6-sulfonic acid) diammonium salt) in the presence of peroxidase (HRP) to form a blue-green product (ABTS^2−^) that can be detected with UV-visible spectrophotometry (λabs = 418 nm). On the other hand, the invertase activity was determined through the quantification of the glucose produced by invertase in the presence of sucrose. Glucose was quantified by the absorbance monitorization of its reaction with 3,5-dinitrosalicylic acid at λ = 540 nm using UV-vis spectroscopy. 

### 2.7. Communication Experiments

Mixtures of 3 mg/mL J1c and J2c, respectively, were prepared in an aqueous solution (20 mM Na_2_SO_4_, pH 7.5). Saccharose (50 mM) was added as substrate of invertase, and the mixture was allowed to react under magnetic stirring at room temperature. The absorbance at 454 nm was measured in time intervals after centrifugation. In parallel, the collected signals were compared to control solutions without saccharose. 

## 3. Results and Discussion

Scheme 1 illustrates the sucrose-responsive intercommunicated Janus nanoparticles system. The enzymes invertase and GOD were anchored on the Au surface, and N-acetyl-L-cysteine and [Ru(bpy)_3_]^2+^ were loaded as cargoes into J1c and J2c, respectively. After the addition of sucrose to a mixture of nanoparticles, invertase from J1c hydrolyzed sucrose to fructose and glucose. In turn, the glucose was converted into gluconic acid by GOD from J2c, producing a pH drop. The local pH drop induced the opening of the pH-responsive β-cyclodextrin-based supramolecular nanovalve on the MSNPs face of J2c, and, consequently, the N-acetyl-L-cysteine was released. Finally, the N-acetyl-L-cysteine previously released provoked the cleavage of the disulfide bond on the MSNPs face from J1c, resulting in the pore opening and [Ru(bpy)_3_]^2+^ delivery, which was monitored by absorbance measurements. 

### 3.1. Characterization

**Figure 1:**
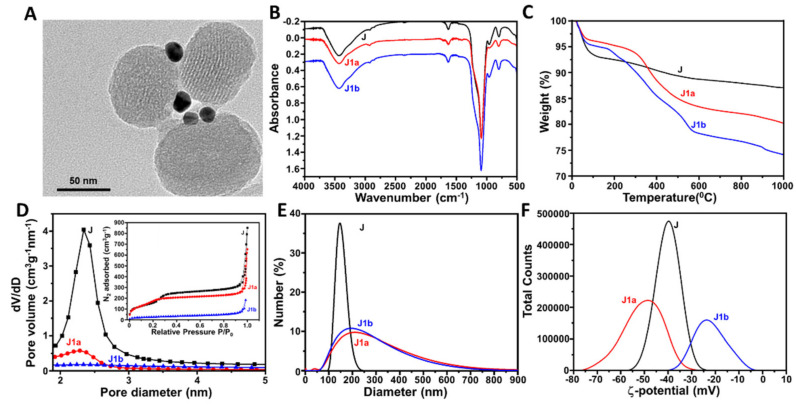
Characterization of the J1c nanodevice: (**A**) HR-TEM image of J. (**B**) Infrared spectra and (**C**) thermograms for the Janus Au-MS nanoparticles J, J1a, and J1b. (**D**) N_2_ adsorption-desorption isotherms for J, J1a, and J1b (Inset: pore size distribution). (**E**) Hydrodynamic diameter and (**F**) Zeta potential of J, J1a, and J1b.

**Figure 2:**
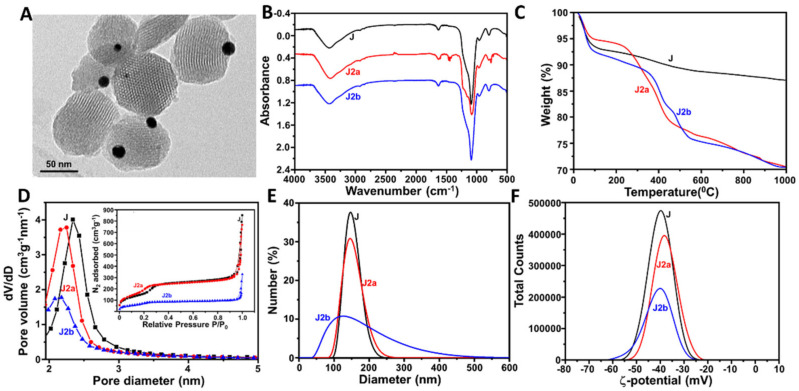
Characterization of the J2c nanodevice: (**A**) HR-TEM image of J. (**B**) infrared spectra and (**C**) thermograms for the Janus Au-MS nanoparticles J, J2a, and J2b. (**D**) N_2_ adsorption-desorption isotherms for J, J2a, and J2b (Inset: pore size distribution). (**E**) Hydrodynamic diameter and (**F**) Zeta potential of J, J2a, and J2b.

The preparation of J1c and J2c is represented in Schemes S1 and S2 (Supplementary Materials). The morphology of J (average diameter: 106 ± 15 nm), which integrated MSNPs and AuNPs, was confirmed by HR-TEM (Figure 1 and Figure 2A). TEM images also showed the porous structure of the MSNPs and the ratio between MSNPs and AuNPs (1:1). The characteristic reflection peak at 2Θ = 2.5 from mesoporous structure throughout the J1c and J2c preparation reinforced that the surface functionalization and cargo loading processes did not damage the mesoporous scaffolding (Figures S1A and S2A). Furthermore, at high angles, the powder X-ray diffractogram (Figures S1B and S2B) for the Janus Au-MS Nanoparticles showed the characteristic cubic AuNPs peaks (2Θ = 37°, 45°, 65°, and 78°), confirming the Janus structure observed through TEM. 

The FT-IR spectra confirmed the functionalization carried out on Janus Au-MS Nanoparticles. In Figure 1B, the presence of the IR absorption band at 1720 cm^−1^ is characteristic of C=O from carboxylic acid moieties in the J1a particle, confirming the successful functionalization of the Au surface with 3-mercaptopropionic acid. β-Cyclodextrin moieties in J1b could not be clearly observed because the broad band at ca. 1060 cm^−1^ [20], which is characteristic for this cyclic oligosaccharide. The overlaps with the characteristic silica signals at 1079–1088 cm^−1^ can be attributed to the asymmetric vibrations of (Si-O-Si), while those at 952–953 cm^−1^ can be attributed to the asymmetric vibrations of (Si-OH) [21]. In Figure 2B, the characteristic IR absorption bands of benzimidazole can be clearly observed in the spectrum of J2a. Thermogravimetric studies on the J, J1a, and J1b (Figure 1C), as well as the J2a and J2b (Figure 2C), revealed a loss of % mass, while the temperature increased due to the degradation of organic molecules anchored or encapsulated in these structures. Moreover, as expected, these results confirmed the efficient encapsulation of [Ru(bpy)_3_]^2+^ (J1b) and N-acetyl-L-cysteine (J2b), respectively, as well as the successful anchoring of the respective nanovalves: sulfonate-β-cyclodextrin (J1b) and the benzimidazole, along with β-cyclodextrin (J2b). 

N_2_ adsorption-desorption isotherms indicate the empty pores on the MS face from the Janus Au-MS Nanoparticles using Brunauer–Emmett–Teller analysis (BET). As observed, both Figure 1 and Figure 2D showed a decrease of N_2_ adsorbed after cargo loading, with this decrease being greater after the incorporation of the supramolecular gates. This technique was also used to estimate the pore diameter of the Janus Au-MS Nanoparticles as 2.4 nm. Similar results were obtained for both particles for J1a and J2b. However, BET analysis evidenced the lowest loading efficiency of N-acetyl-L-cysteine in J2a in comparison with [Ru(bpy)_3_]^2+^ in J1a. This can be attributed to the fact that, unlike [Ru(bpy)_3_]^2+^, N-acetyl-L-cysteine, was soluble in the solvent employed for the anchoring of the nanovalve, which provoked an undesired release of the cargo. 

Each step of the preparation of J1c (Figure 1E,F) and J2c (Figure 2E,F) was also monitored using dynamic light scattering (DLS). Figure 1E displays wider peaks and, thus, an increase of the hydrodynamic diameter after functionalizing the Au and MS faces. This can be attributed to the emergence of Van der Waals forces between thiol moieties and Au faces, which generates aggregates with a variable number of particles being the average diameters of J1a and J1b, 209 ± 154 nm and 240 ± 191 nm, respectively. As expected, the diameter of J1b was larger than J1a because of the presence of CD on the MS face of J1b. On the contrary, for the preparation of J2c, the aggregates of particles were not observed, since the functionalities added on the MS face of J2a did not interact with the Au surfaces of other particles (Figure 2E). However, as expected, once CD was added to close the nanovalve, the hydrodynamic diameter increased (120 ± 80 nm). Furthermore, the Z potential confirmed the success of each step in the preparation of each particle. Figure 1F illustrates more negative Z potential values for J1a (−48 ± 21 mV) because of the presence of thiol and carboxylic acid moieties on the MS and Au faces, respectively, which agree with the literature [22,23]. Nevertheless, after CD anchored to form J1b, the Z potential shifted to more positive values, but remained negative (−23 ± 20 mV). Different behavior was observed for the preparation of J2c (Figure 2F), whose precursor particles showed similar Z potential values throughout their surface modifications. This could be ascribed to the presence of positively charged benzimidazole moieties on the mesoporous face, thus compensating the negative charge of the carboxylic acid groups at the Au face on J2a and J2b. 

Finally, the quantity and specific activity of the anchored proteins were estimated using the Bradford and specific activity assays, respectively. On the one hand, the invertase anchored on the Au-face of J1c was 14.5 µg mg^−1^ and presented an activity of 1.07 U mg^−1^, which was calculated through the quantification of the formed sugar molecules (D-glucose and D-fructose). On the other hand, the GOD anchored on the Au-face of J2c was 27.9 µg mg^−1^ and presented an activity of 0.86 U mg^−1^, which was calculated through the ABTS/HRP assay. The efficiency of cargo loading was also estimated for both nanosystems. The results showed a higher efficiency of encapsulation for [Ru(bpy)_3_]^2+^ (12 µmol g^−1^ of particles) in J1c in comparison with N-acetyl-L-cysteine (30 µmol g^−1^ of particles) in J2c.

### 3.2. Kinetic Release Assays

**Figure 3:**
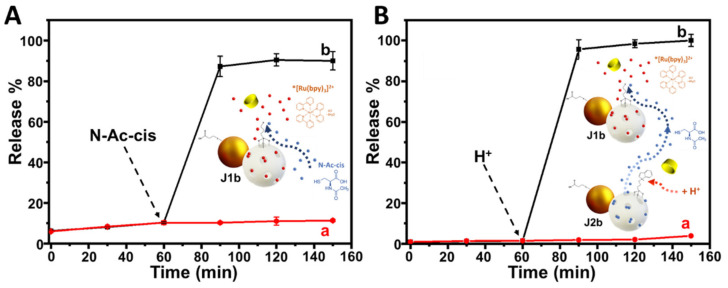
Cargo release assays control assays: (**A**) Kinetics of [Ru(bpy)_3_]^2+^ release encapsulated in J1b in the presence of 50 mM N-acetyl-L-cysteine. (**B**) Kinetics of [Ru(bpy)_3_]^2+^ release encapsulated in J1b in the presence of J2b and the addition of acid media. (Conditions: 20 mM Na_2_SO_4_, pH 7.5, 25 °C, [J1b] = 3 mg mL^−1^, [J2b] = 3 mg mL^−1^, λ_abs_ = 454 nm). Error bars correspond to the s.d. from three independent experiments.

To demonstrate that the J1c and J2c nanosystems worked correctly, different kinetic release assays were first carried out for the individual evaluation of the cyclodextrin-gated MS face of both nanosystems without enzyme control (Figure 3). As it can be observed in Figure 3A, the addition of N-acetyl-L-cysteine at the 50 mM final concentration at t = 60 min in an aqueous solution containing J1b particles induced the release of [Ru(bpy)_3_]^2+^. This release was monitored by UV-Vis, confirming the appropriate performance of the N-acetyl-L-cysteine-responsive supramolecular nanovalve. Next, to probe the correct performance of the pH-responsive supramolecular nanovalve, an aqueous mixture of both nanosystems, J2b and J1b, was adjusted to an acidic pH by adding hydrochloric acid until reaching a final concentration of 0,67 M. This pH change caused the breakdown of the interactions between benzimidazole and β-cyclodextrin. Consequently, the interaction breakdown led to the release of N-acetyl-L-cysteine encapsulated in J2b, which, in turn, provoked the total release of [Ru(bpy)_3_]^2+^ from J1b in less than 30 min (Figure 3B). In both cases, control assays showed that without the INPUT molecules, the responsive nanovalve did not undergo any transformation. Thus, the [Ru(bpy)_3_]^2+^ was not released over time.

**Figure 4:**
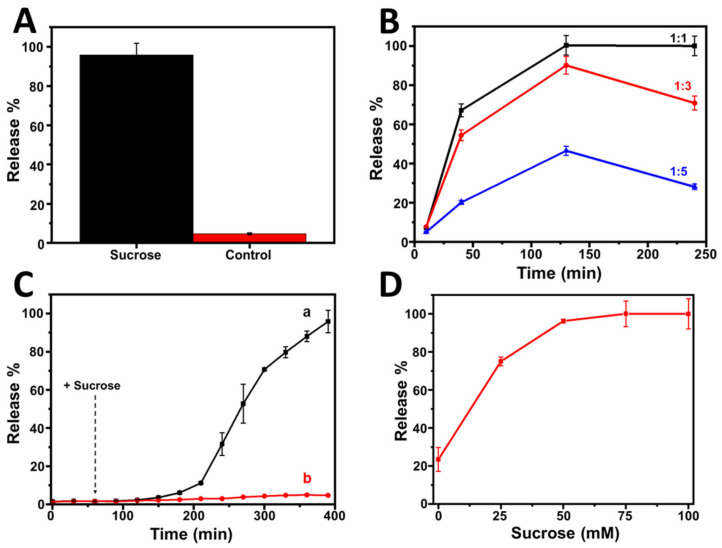
Sucrose-responsive interaction between Janus nanoparticles: (**A**) % of [Ru(bpy)_3_]^2+^ release encapsulated in J1c because of the interaction between Jc1c and Jc2c after 100 min of sucrose (50 mM) addition. (**B**) Kinetics of [Ru(bpy)_3_]^2+^ release encapsulated in J1c for different proportions of J2c in the presence of 50 mM of sucrose. (**C**) Kinetics of [Ru(bpy)_3_]^2+^ release encapsulated in J1c because of the interaction between J1c and J2c after the addition of 50 mM of sucrose. (**D**) % of [Ru(bpy)_3_]^2+^ release encapsulated in Jc1c because of the interaction between J1c and J2c at different sucrose concentrations (t = 6h). (Conditions: 20 mM Na_2_SO_4_, pH 7.5, 25 °C, [J1b] = 3 mg mL^−1^, [J2b] = 3 mg mL^−1^, λabs = 454 nm). Error bars correspond to the s.d. from three independent experiments.

Once the proper operation of the nanovalves was confirmed, the chemical response generated by the communication system established between both nanosystems, J1c and J2c, was evaluated using sucrose (50 mM) as the INPUT chemical signal. Figure 4A displays the % of [Ru(bpy)_3_]^2+^ released after 100 min of sucrose addition to a mixture of the same concentration of both nanosystems (3 mg mL^−1^). It can be observed that most of the cargo loaded in J1c was released, demonstrating the good performance of the sucrose-responsive- intercommunicated Janus nanoparticles network. To optimize the communication between both nanosystems, we studied the ratio between J1c and J2c. To this end, the J1c nanosystem remained constant, and the J2c nanosystem varied, presenting the ratio with the best performance of the Janus network, 1:1 (Figure 4B). The kinetics studies revealed that more than 6 h were required to reach the maximum release of [Ru(bpy)_3_]^2+^ from J1c after the addition of 50 mM of sucrose to a mixture of J1c and J2c under the optimized conditions (3mg mL^−1^ of each nanosystem) (Figure 4C). This slow profile release was due to the involvement of two enzymatic phases prior to the release process. As expected, without the addition of sucrose, no release was observed over the time, which confirmed the nonactivation of the system and, thus, the absence of communication between both nanosystems. The performance of the sucrose-responsive intercommunicated Janus nanoparticles network was evaluated at different sucrose concentrations (0–100 mM). It was observed that as the sugar concentration increased, the release improved until reaching its maximum at 50 mM (Figure 4D).

**Figure 5:**
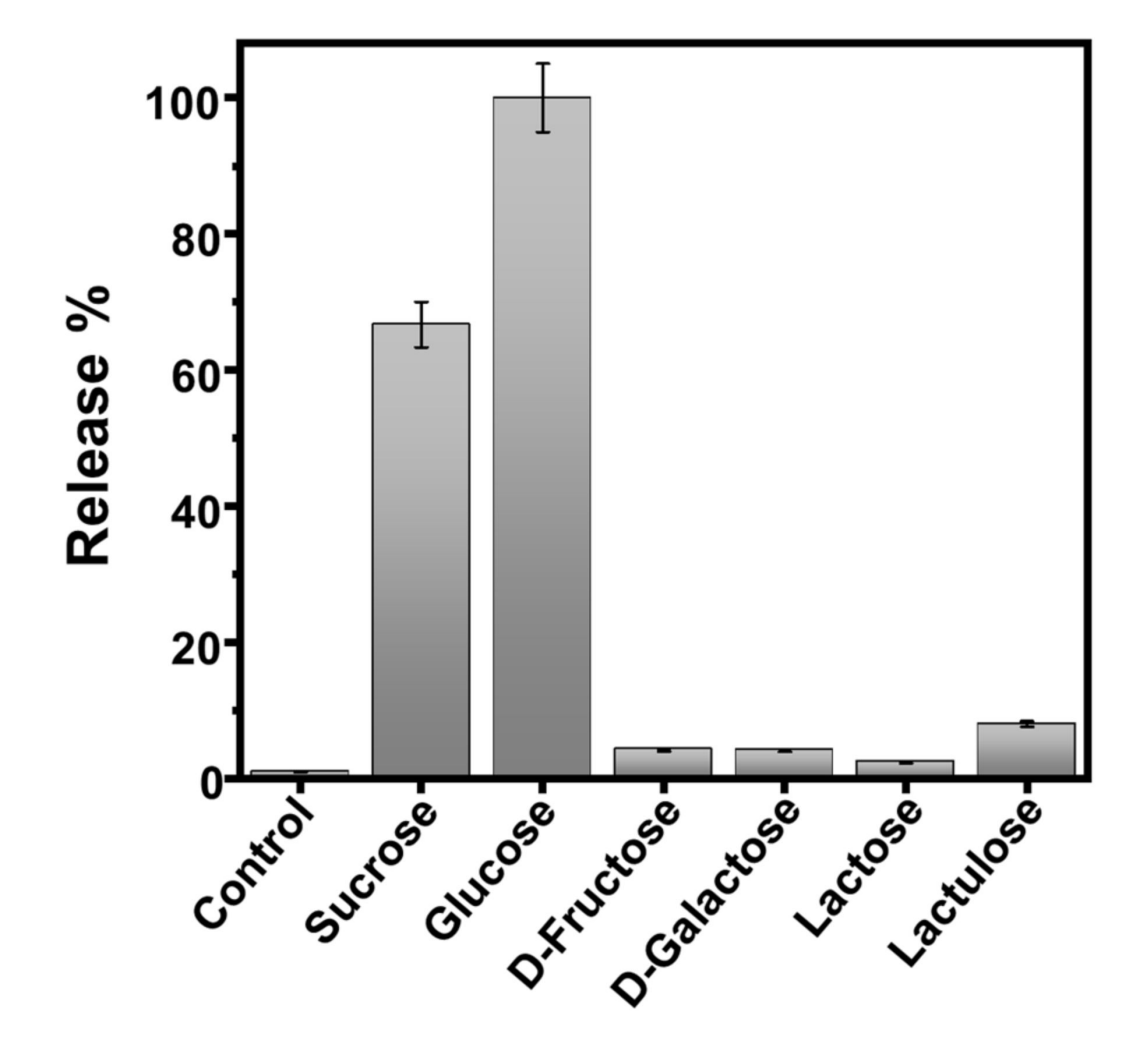
Effect of the presence of different sugars to the communication system’s established chemical response. (Conditions: 20 mM Na_2_SO_4_, pH 7.5, 25 °C, [J1c] = 3 mg mL^−1^, [J2c] = 3 mg mL^−1^, λabs = 454 nm). Error bars correspond to the s.d. from three independent experiments.

Finally, the selectivity of the sucrose-responsive intercommunicated Janus nanoparticles network was analyzed against other sugars. To this end, both nanosystems, J1c and J2c, were mixed (1:1), and the release of [Ru(bpy)_3_]^2+^ was monitored at time 350 min after the addition of glucose, D-fructose, D-Galactose, lactose, or lactulose, respectively (Figure 5). As expected, the results showed that the system was selective for most of the sugars employed. When glucose was used as the INPUT, the release was 30% more efficient than when sucrose was used. This can be attributed to the process that activates glucose, which is mediated only by one enzyme, GOD. In contrast, the process that activates sucrose is mediated by two enzymes, invertase and glucose oxidase, negatively affecting the kinetics of the fabricated system.

## 4. Conclusions

In summary, we developed a new example of communication between two enzyme-controlled nanodevices based on Janus Au-mesoporous silica using sucrose as an INPUT signal. The sucrose enzymatic-based sensing in one of the nanodevices successfully triggered the sequential delivery of two different molecules, N-acetyl-L-cysteine and [Ru(bpy)_3_]^2+^. This work opens new possibilities for the development of intercommunicated nanodevices able to provoke a selective and sequential release of target molecules. Therefore, these systems are a potential tool in several fields, such as biomedicine for disease treatment.

## Supplementary Materials

The following are available online at https://www.mdpi.com/article/10.3390/nano11102492/s1, Materials and methods: Synthesis of mono-6- Iodine-desoxi cyclodextrin (β-CD-I) and Synthesis of methanethiosulfonate de 5-(6-desoxi-β-cyclodextrin) (β-CD- SSO_2_CH_3_). Scheme S1: Preparation steps for J1c., Scheme S2: Preparation steps for J2c. Figure S1: Powder X-ray diffraction patterns of the solids J, J1a y J1b at low (A) and high (B) angles. Figure S2: Powder X-ray diffraction patterns of the solids J, J2a y J2b at low (A) and high (B) angles.
