# Supplementary material for: Sucrose-Responsive Intercommunicated Janus Nanoparticles Network
Source: Nanomaterials (Basel). 2021 Sep 24;11(10):2492. doi: 10.3390/nano11102492 (PMC8540595; doi:10.3390/nano11102492)
Supplement: Supplementary file 1 [file nanomaterials-11-02492-s001.zip › nanomaterials-1360816 suplementary.pdf]

Supplementary Information

Sucrose-responsive Intercommunicated Janus Nanoparticles Network

Sandra Jimenez-Falcao,[1] Daniel Torres,[1] Paloma Martínez-Ruiz,[1] Diana Vilela,[1] Ramón Martínez-Máñez,[2,3,4] and Reynaldo Villalonga[1*]

[1] Nanosensor & Nanomachines Group, Department of Analytical Chemistry, Faculty of Chemistry, Complutense University of Madrid, 28040-Madrid, Spain.
[2] Instituto Interuniversitario de Investigación de Reconocimiento Molecular y Desarrollo Tecnológico (IDM), Universitat Politècnica de València, Universitat de València, Camino de Vera s/n, 46022, Valencia, Spain.
[3] Unidad Mixta UPV-CIPF de Investigación en Mecanismos de Enfermedades y Nanomedicina, Universitat Politècnica de València, Centro de Investigación Príncipe Felipe, Valencia, Spain.
[4] CIBER de Bioingeniería, Biomateriales y Nanomedicina (CIBER-BBN), Spain.
* Correspondence: rvillalonga@quim.ucm.es

1. Materials and Methods

1.1 Ensemble of the nanomachine: J1c

1.1.2 Synthesis of mono-6- Iodine-desoxi cyclodextrin (β-CD-I)

For the synthesis of mono-6- Iodine-desoxi cyclodextrin (β-CD-I), previously reported methods have been used.[1,2] Briefly, 1.5 g (1 mmol) of mono-6-O-(p-toluenesulfonyl)-beta-cyclodextrin (β-CD-Ts) previously synthesized[3] and 1.75 g (12 mmol) of NaI are disolved in anhydrous DMF under inert atmosphere. The mix is heated under reflux with magnetic stirring for 5 hours. Then, the solvent is removed under reduced pressure and the product precipitates as a white solid after the addition of 50 mL of acetone. The solid product is filtered and washed several times with acetone and finally is dissolved in the least amount of water to, subsequently, precipitate it again with acetone. Finally, the solid obtained, β-CD-I, is vacuum filtered and washed with acetone being storage under vacuum.

The resulting yield was: *1.24 g, 87 %.*

1.1.2 Synthesis of methanethiosulfonate de 5-(6-desoxi-β-cyclodextrin) (β-CD- SSO_2CH_3)

For the synthesis of methanethiosulfonate de 5-(6-desoxi-β-cyclodextrin) (b-CD- SSO_2CH_3), previously reported methods have been employed.[1,2] Briefly, 0.5 g of β -CD-I (0.4 mmol) and 72 mg of $NaSSO_2CH_3$ (0.5 mmol) were dissolved in 5 mL of DMF under Ar atmosphere using a 25 mL round bottom flask with two necks. The mix was stirred and heated at 50 °C for 24 hours. Afterwards, the solvent is removed under reduced pressure and the product precipitates as a white solid after the addition of ethanol. The solid is vacuum filtered and washed several times using a filter plate being storage under vacuum.
The resulting yield was: *0.41 g, 82 %.*

1.2 Schematic representation of the preparation of both nanomachines: J1c and J2c

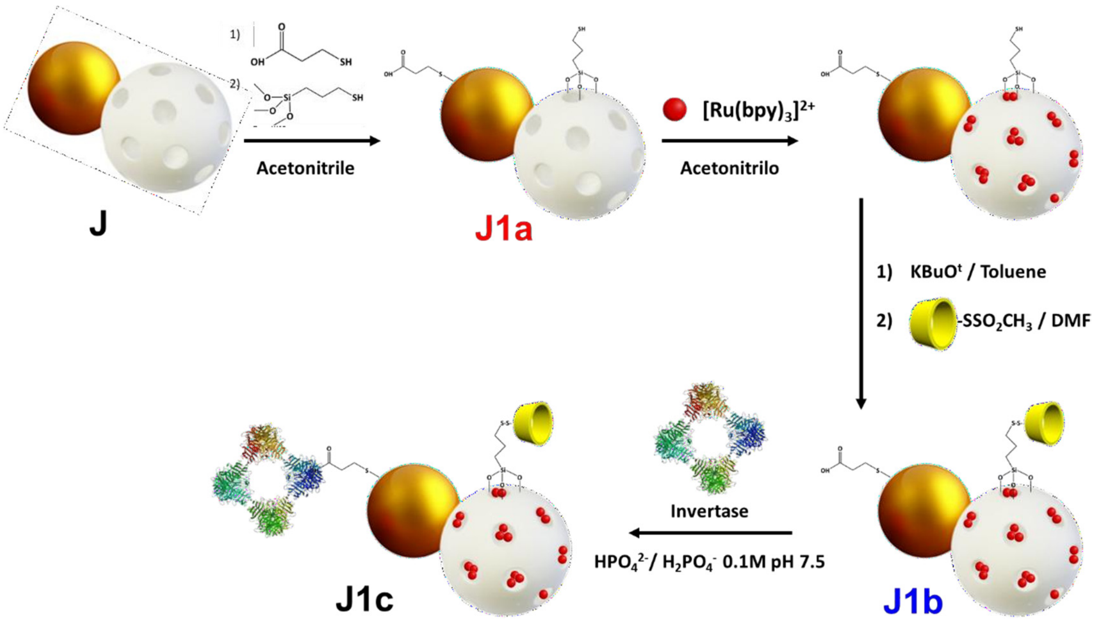

Scheme S1. Preparation steps for J1c.

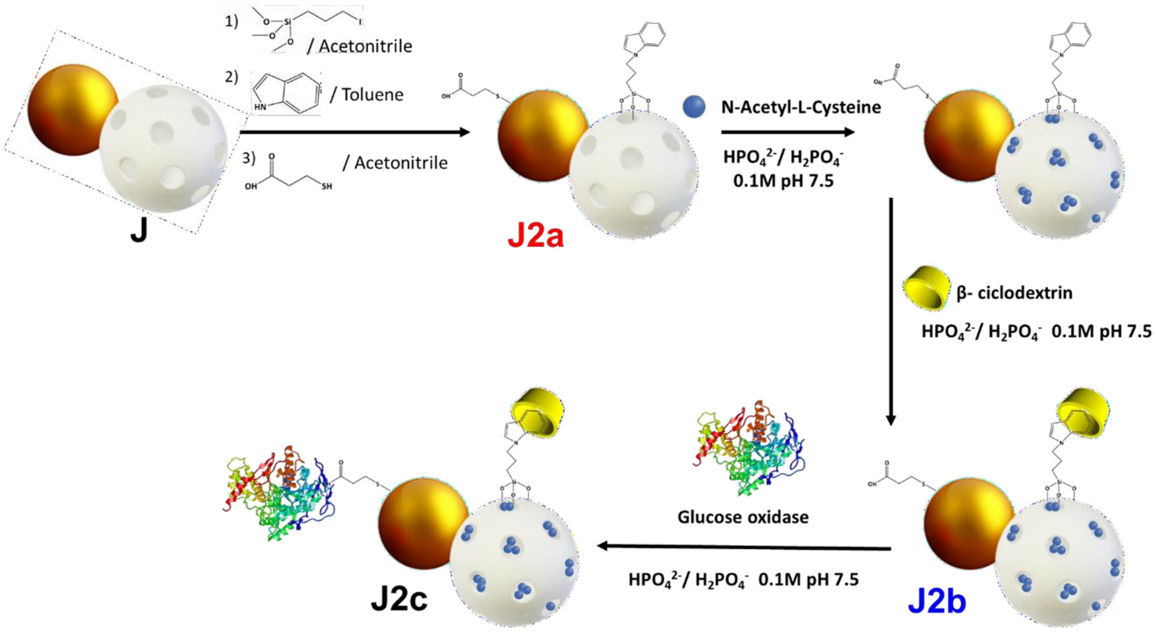

Scheme S2. Preparation steps for J2c.

2. Results and Discussion

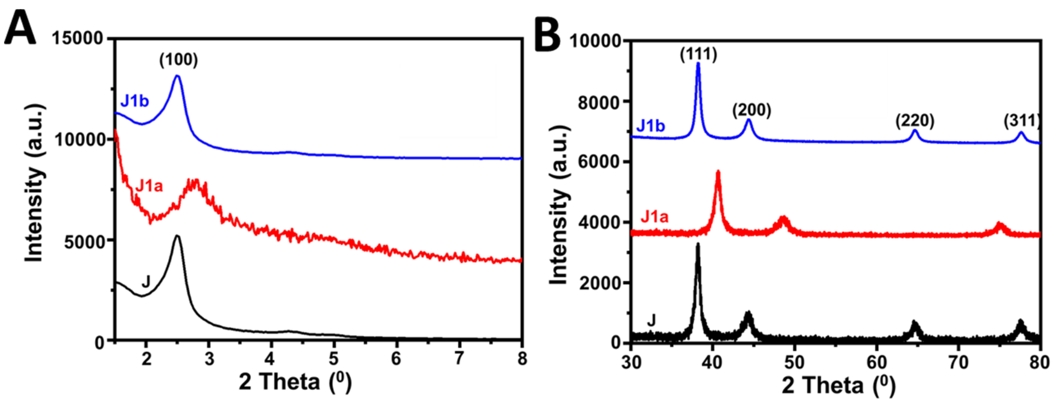

Figure S1. Powder X-ray diffraction patterns of the solids J, J1a yJ1b at low (A) and high (B) angles.

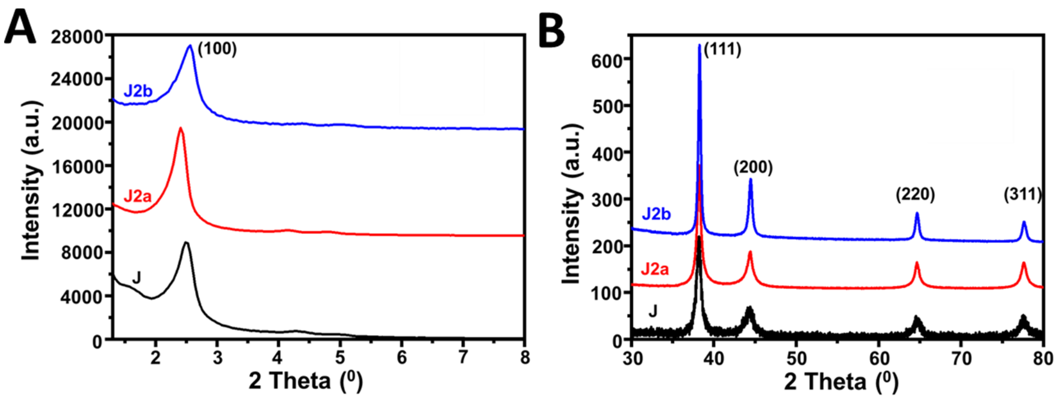

Figure S2. Powder X-ray diffraction patterns of the solids J, J2a y J2b at low (A) and high (B) angles.
